# Improving wheat grain yield via promotion of water and nitrogen utilization in arid areas

**DOI:** 10.1038/s41598-021-92894-6

**Published:** 2021-07-05

**Authors:** Yan Tan, Qiang Chai, Guang Li, Cai Zhao, Aizhong Yu, Zhilong Fan, Wen Yin, Falong Hu, Hong Fan, Qiaomei Wang, Yao Guo, Xuemei Tian

**Affiliations:** 1 College of Forestry, Gansu Agricultural University, Lanzhou, 730070 China; 2 Gansu Provincial Key Laboratory of Aridland Crop Science, Lanzhou, 730070 China; 3 College of Agronomy, Gansu Agricultural University, Lanzhou, 730070 China

**Keywords:** Plant sciences, Systems biology

## Abstract

Crop yield is limited by water and nitrogen (N) availability. However, in Hexi Corridor of northwestern China, water scarcity and excessive fertilizer N in wheat (*Triticum aestivum* L.) production causes serious conflicts between water and N supply and crop demand. A field experiment was conducted from 2016 to 2018 to evaluate whether reducing of irrigation and fertilizer N will reduce grain yield of wheat. There were two irrigation quotas (192 and 240 mm) and three fertilizer N rates (135, 180, and 225 kg N ha^−1^). The results showed that reducing irrigation to 192 mm and N rate to 180 kg N ha^−1^ reduced water uptake, water uptake efficiency, and N uptake of spring wheat as compared to local practice (i.e., 240 mm irrigation and 225 kg N ha^−1^ fertilizer). Whereas, it improved water and N utilization efficiency, and water and N productivity. Consequently, the irrigation and N rate reduced treatment achieved the same quantity of grain yield as local practice. The path analysis showed that interaction effect between irrigation and N fertilization may attributable to the improvement of grain yield with lower irrigation and N rate. The enhanced water and N utilization allows us to conclude that irrigation quota at 192 mm coupled with fertilizer N rate at 180 kg N ha^−1^ can be used as an efficient practice for wheat production in arid irrigation areas.

## Introduction

The growing of human population with dwindling natural resources has made agriculture facing with unprecedented challenges^1^. Wheat (*Triticum aestivum* L.), the third most important crop, is widely grown across the world to provide sufficient quantities of food^2^. In Hexi Corridor of northwestern China, the most important commodity grain bases area, wheat is largely adopted^3^. This region has annual mean pan evaporation greater than 2000 mm, leading to serious water shortage^4^. Due to crop water requirement is much greater than precipitation, wheat production relies heavily on irrigation^5^. However, the farmers normally use unreasonable levels of irrigation and N fertilizer supplementation in an attempt to increase yields^6^. This not only reduced water and fertilizer use efficiency, but also increased the risk of resource wasting^7^. Thus, more effective water and N fertilizer management strategies are urgently required. Numerous studies have been conducted formerly to optimize the irrigation and N fertilization^8,9^. However, there still remaining unknown of the underlying mechanism on coordinating use of water and nitrogen.

Water productivity, an indicator of water use efficiency, is primarily used to evaluate if a particular practice can improve grain yield with less water. Some researchers suggested that any efforts to increase water productivity should focus on yield improvement, and the associated agronomic approaches including tillage options, cropping systems, and fertilizer rates^10–12^. While others concluded that reducing evapotranspiration can improve water productivity^13–15^. Therefore, improving N fertilizer rate to increase transpiration and/or lowering irrigation to reduce soil evaporation become a key to improve water productivity^16,17^.

Nitrogen use efficiency is an important indicator for assessing N productivity^18,19^, which consisting of N uptake efficiency and utilization efficiency^20^. Therefore, optimizing N uptake and utilization of a crop is important to achieve the higher N productivity^18^. It has been found that irrigation and N fertilization are two vital factors influencing N uptake and utilization^21^. Irrigation often increases N utilization through the improvement of N translocation, distribution and accumulation^22,23^. However, N fertilization increases N uptake but reduces N utilization^24,25^. Many researches have confirmed that N productivity strongly relied on N utilization but not N uptake^26–28^. Accordingly, reducing N fertilizer rate and improving irrigation are essential measures to improve the N productivity.

It is contradictory to improve water and N productivity at the same time with the single management of irrigation or N fertilization. It has been found that irrigation improves grain yield mainly through N productivity, while N fertilization modifies grain yield through water productivity^21^. This implies an interaction-effect between irrigation and N fertilization. Therefore, integration of irrigation and N fertilization are fundamentally required in current wheat production. In order to quantitively analysis the interaction-effect between irrigation and N fertilization, a field experiment with various ratios of irrigation quota and fertilizer N rate were combined and compared. The primary objective of this study was to evaluate how irrigation and fertilizer N combination will influence the water and N productivity of spring wheat. We hypothesized that water and N productivity of wheat could be enhanced through the improvement of water and N utilization efficiency. In testing the hypothesis, we determined (1) grain yield (2) water and N uptake, and (3) water and N use efficiencies.

## Materials and methods

### Experiment site

**Figure 1:**
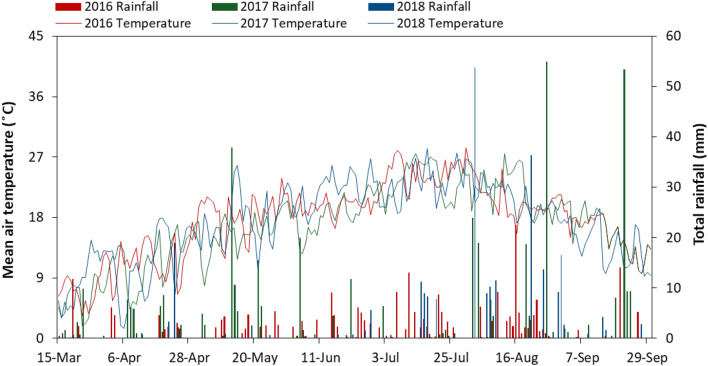
Mean air temperature and rainfall during the growing season after harvesting of spring wheat in 2016, 2017, and 2018 at Wuwei experimental station, northwestern, China.

The experiment was conducted at the Oasis Agricultural Experimental Station of Gansu Agricultural University (Gansu Province, China; 37° 30′ N, 103° 5′ E; 1776 m a.s.l.) in 2016–2018. This station is located in the eastern part of the Hexi Corridor of northwestern China. The long-term average annual precipitation is 160 mm, with two-thirds of that falls between July and September (Fig. 1), and the potential evaporation is greater than 2000 mm. The annual temperature is 7.2 °C, with accumulated temperature above 0 °C of > 3513 °C and above 10 °C of > 2985 °C, and a frost free period of 156 d. The soil at the experimental site is classified as an Aridisol^29^, with 8.0 pH (1:2.5 soil:water), 14.3 g kg^−1^ OC, 0.78 g kg^−1^ total N, 1.76 mg kg^–1^ NH_4_^+^–N and 12.3 mg kg^–1^ NO_3_^−^–N prior to the start of the experiment. The soil bulk density in 0–110 cm soil depth averages 1.44 g cm^−3^ (Table 1). Agriculture in this region depends greatly on irrigation and fertilization. Whereas, water scarcity increasingly threatening the agriculture in recent years.

**Table 1:** Main soil properties across 0–100 cm soil profile at the Oasis Agricultural Experimental Station, China.

Soil depth (cm)	Wilting point (%)	Filed capacity (%)	Bulk density (g cm^−3^)	Soil texture^a^	Particle size (%)^b^		
					Sand	Silt	Clay
0–20	6.9	20.2	1.41	Silt loam	28.6	65.4	5.1
20–40	9.6	23.4	1.45	Silt loam	25.6	69.8	4.6
40–70	10.2	26.2	1.43	Silt	16.7	79.8	4.1
70–100	11.4	27.6	1.47	Silt loam	25.6	70.2	3.6

^a^Soil texture was determined according to soil particle percentage.

^b^Soil particle fraction was determined based on the USDA textural soil classification system.

### Experimental design and crop management

The experiment utilized a split plot arrangement of treatments in a randomized complete blocks design. There were three replicates for each treatment. The main plot factor was irrigation quota, consisting of 192 mm (I1) and 240 mm (I2, the local practice); and subplot factor was fertilizer N rate, consisting of 135 kg N ha^−1^ (N1), 180 kg N ha^−1^ (N2), and 225 kg N ha^−1^ (N3, the local practice). The plot size was 10 × 5.5 m^2^, with a 0.8 m wide by 0.5 m high ridge between adjacent plots to eliminate potential movement of irrigation water. For I1 treatment, each plot received 60, 72, and 60 mm of irrigation water at wheat seedling, booting, and grain filling stage respectively, while in I2 treatment, each plot received 75, 90, and 75 mm of irrigation water at three stages. No matter in I1 or I2 treatment, all plots received 120 mm of irrigation in late fall just before soil freezing (Table 2). A hydrant pipe system was used for irrigation and flow meters were used to record the irrigation volume applied in each plot. For N fertilization, urea (46–0–0, N–P–K) with respective N rate were broadcast and incorporated into the soil prior to seeding as base fertilizer. In conjunction with N fertilizer application, all plots received a base application of phosphate fertilizer as calcium superphosphate at (0–16–0 of N–P–K) at 100 kg P ha^−1^. Wheat (cv. Long-chun 30) was sown in late March and harvested in late July in 2016, 2017 and 2018. The row spacing was 15 cm and plant density was 4,650,000 plants ha^−1^.

**Table 2:** Supplemental irrigation dates and quotas at the main growth stages of wheat, at the Oasis Agricultural Experimental Station, with the same schedule and amount being used for each of the experimental years.

Irrigation schedule	Irrigation date			Irrigation quota (mm)	
	2016	2017	2018	I1	I2
Late fall	28-November	3-December	25-November	120	120
Seedling	5-May	3-May	2-May	75	60
Booting	7-June	28-May	9-June	90	72
Grain filling	28-June	23-June	23-June	75	60

### Measurement and calculation

#### Grain yield

Grain yield (GY) was assessed by each plot when wheat reached full maturity. After threshing, cleaning, and air-drying, the gains were weighed for recording of the GY.

#### Water uptake

The total evapotranspiration (ET, mm) consisted of transpiration (T, mm), soil evaporation (SE, mm), and canopy evaporation (CE, mm) of field crops^16^. However, in this region, CE was negligible due to low precipitation, especially in wheat growing season (Fig. 1). Therefore, the water uptake (W-uptake, mm), which defined as water consumed by crop plants^17^, was equal to T and calculated as:

1 $${\text{W-uptake}} = {\text{ET}} - {\text{SE}}$$

The ET was determined using the water balance equation as follows:

2 $${\text{ET}} = P + I + U - R - DW - \Delta S$$

where P is precipitation during the growing season (mm), I is the amount of irrigation (mm), U is upward capillary flow from the root zone (mm), R is runoff (mm), DW is downward drainage out of the root zone (mm), and ΔS is the change of soil water storage in the 0–120 cm layer (mm) before planting and after harvesting. The upward capillary flow and downward drainage out of the root zone were negligible in this area according to Xie et al.^30^. Runoff was also negligible due to small rains. Therefore, ET was the sum of precipitation, irrigation and the change in soil water storage.

For the determination of soil water storage, the soil water content was firstly measured. A frequency of 20 days during the entire growing season was applied for the measurement. The soil water content at 0–30 cm depth by 10 cm increments were measured using the oven-drying method, while at 30–120 cm depth by 30 cm increments were measured using neutron probe (NMM 503 DR, USA) according to Yin et al.^31^.

The SE in this study was determined by using the micro-lysimeters^30^. They were constructed using polyvinyl chloride tubes with a length of 15 cm, an internal diameter of 11.5 cm, and an external diameter of 12 cm. The base of the tubes was sealed with waterproof tape. A micro-lysimeter was placed in the center of each plot. Each micro-lysimeter was filled with soil and placed into a larger (12-cm internal diameter) polyvinyl chloride tube that was previously installed in the field^31^. All micro-lysimeters were weighed at 18:00 at a 3–5-d interval from planting to harvest using a portable electronic balance. The SE was recorded and calculated from the weight loss between two measurements (1-g change was equivalent to 0.1053-mm of SE).

#### Nitrogen uptake

At wheat maturity, a 20 cm length of 6 rows of wheat in each plot were harvested to assess the aboveground dry matter. All the collected plant samples were oven-dried at 105 °C for desiccation and then placed at 80 °C until it reached a constant weight. The samples were separated into leaves, stems and grains thereafter and then milled and mixed thoroughly^32^. The N concentration (%) of wheat samples were measured by a high-induction furnace C and N analyzer (Elementar vario MACRO cube, Hanau, Hessen, Germany). Total aboveground N accumulation, i.e. total N uptake (N-uptake, kg ha^−1^), was calculated as the product of each aboveground organ dry matter and corresponding N concentrations^33^.

#### Water use characteristics

In order to evaluate the proportion of water consumed by crop plants (without soil evaporation) in the total water consumption (i.e. evapotranspiration), we created the term water uptake efficiency (WupE, %), and calculated as:

3 $${\text{WupE}} = {\text{W-uptake/ET}} \times 100\%$$

Water utilization efficiency (WutE, kg ha^−1^ mm), i.e. the yield-to-transpiration ratio^17^, defined as gain yield per mm of water consumed by crop plants, was calculated as:

4 $${\text{WutE}} = {\text{GY/W-uptake}}$$

Water use efficiency (WUE, kg ha^−1^ mm), i.e. water productivity, was the product of WupE and WutE, and defined as gain production per mm of total water consumption^31^. It was calculated as follows:

5 $${\text{WUE}} = {\text{WupE}} \times {\text{WutE}} = {\text{GY/ET}}$$

#### Nitrogen use characteristics

Nitrogen uptake efficiency (NupE, %) was calculated by dividing the total above ground N uptake at harvest by the amount of N available to the crop from soil and fertilizer according to Hawkesford^34^ and Cohan et al.^20^. It was calculated as:

6 $${\text{NupE}} = {\text{N-uptake/}}\left( {\text{N-soil}} + {\text{N-fertilizer}} \right) \times 100\%$$

where N-soil is soil mineral N accumulation across the 0–80 cm soil layer before planting. For this study, the N-soil is 139.8, 149.6 and 134.0 kg N ha^−1^ in 2016, 2017, and 2018, respectively. The soil mineral N accumulation was determined according to Hu et al.^33^ by using a segmented flow injection autoanalyzer (Autoanalyser 3, Bran-Luebbe, Germany). N-fertilizer is the fertilizer N rate at 135, 180 and 225 kg N ha^−1^ for N1, N2, and N3, respectively.

Nitrogen utilization efficiency (NutE, kg kg^−1^) was defined as the grain production per unit of N uptake^20^, and calculated as:

7 $${\text{NutE}} = {\text{GY/N-uptake}}$$

Nitrogen use efficiency (NUE, kg kg^−1^), i.e. nitrogen productivity, was the product of NupE and NutE, and defined as the grain production per unit of N available to the crop from soil and fertilizer^34^. It was calculated as follows:

8 $${\text{NUE}} = {\text{NupE}} \times {\text{NutE}} = {\text{GY/}}\left( {\text{N-soil}} + {\text{N-fertilizer}} \right)$$

### Statistical analysis

The experimental data were analyzed with the statistical analysis software of SPSS 17.0 (SPSS Inc., Chicago, IL, USA). The treatment effects were investigated using the standard split-plot design analysis method. Year, irrigation quota and N fertilizer rate were considered as fixed effects and replication as random effects. Means were compared by least significance difference (LSD). All determinations of significance were declared at the probability level of 0.05. Path analysis was conducted using the stepwise method.

## Result

### Grain yield

**Figure 2:**
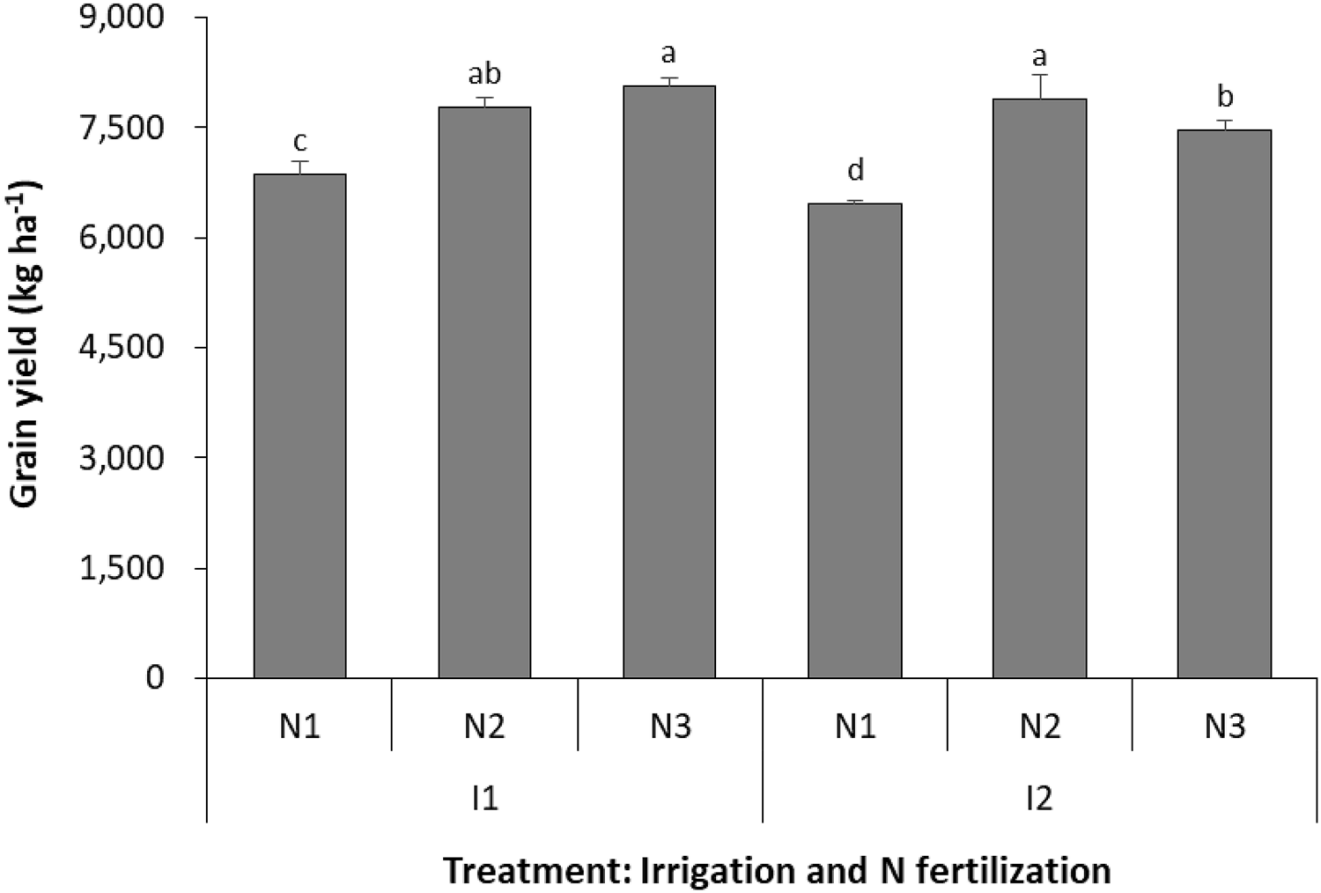
Grain yield of spring wheat with two irrigation quotas and three N fertilizer rates across 2016–2018. I1 and I2 represent irrigation amount at 192 and 240 mm, respectively. N1, N2 and N3 represent an N fertilizer rate of 135, 180, and 225 kg N ha^−1^, respectively. Different letters indicate significant differences (*P* < 0.05) among treatments and the smaller bars are standard error of means (n = 9).

Grain yield (GY) of wheat was significantly affected by irrigation quota (*P* = 0.006), N fertilizer rate (*P* < 0.001), and irrigation quota × N fertilizer rate interaction (*P* = 0.014), but not by year × irrigation quota × N fertilizer rate interaction (*P* = 0.132). At irrigation quota of 190 mm (I1), the GY with N rate of 180 kg N ha^−1^ (N2) and 225 kg N ha^−1^ (N3) were increased by 13.2 and 17.5% compared to N rate of 135 kg N ha^−1^ (N1), respectively (Fig. 2, Table S-1). Similarly, at irrigation quota of 240 mm (I2), the GY with N2 and N3 were increased by 22.3 and 15.7% compared to N1, respectively. Besides, with N1 treatment, the GY at I1 was increased by 6.3%, and with N3 increased by 7.9%, compared to I2. There was no significant difference of GY between two irrigation treatments with N2.

### Water uptake

**Figure 3:**
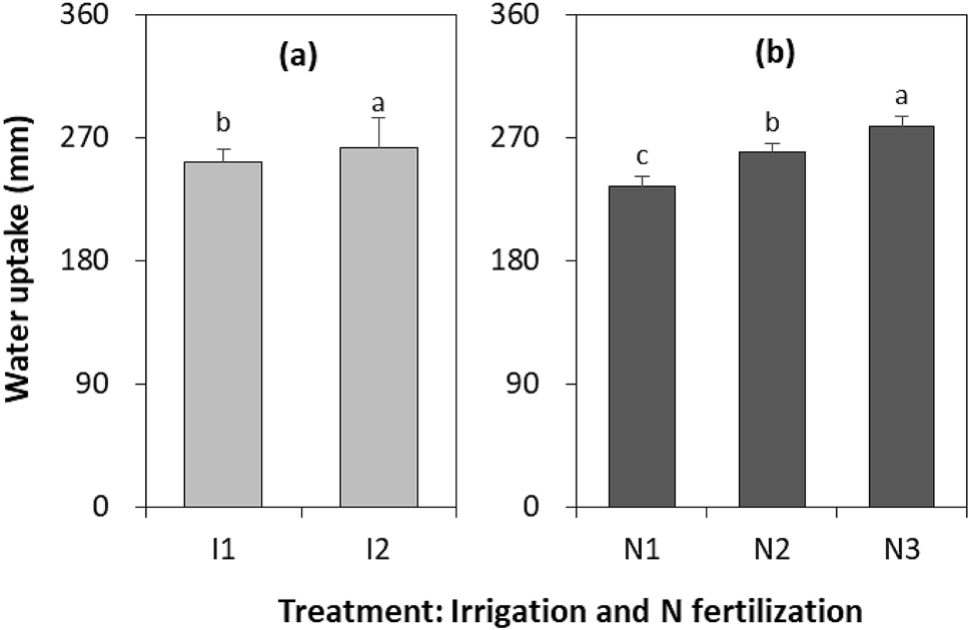
Water uptake of spring wheat during growing season with two irrigation quotas and three N fertilizer rates across 2016–2018. I1 and I2 represent irrigation amount at 192 and 240 mm, respectively. N1, N2 and N3 represent an N fertilizer rate of 135, 180, and 225 kg N ha^−1^, respectively. Different letters indicate significant differences (*P* < 0.05) among treatments and the smaller bars are standard error of means (n = 9).

The effect of irrigation quota × N fertilizer rate interaction (*P* = 0.987) and year × irrigation quota × N fertilizer rate interaction (*P* = 0.707) on W-uptake of wheat were not significant, but irrigation quota (*P* < 0.001) and N fertilizer rate (*P* < 0.001) individually affected it (Fig. 3, Table S-1). Compared to I2, the W-uptake with I1 was reduced by 3.9%. Compared to N1, N2 and N3 increased the W-uptake by 10.3 and 18.7%. While, N2 reduced W-uptake by 7.0% as compared to N3. This indicates that reducing N fertilizer rate reduces water uptake from soil layers.

### Nitrogen uptake

**Figure 4:**
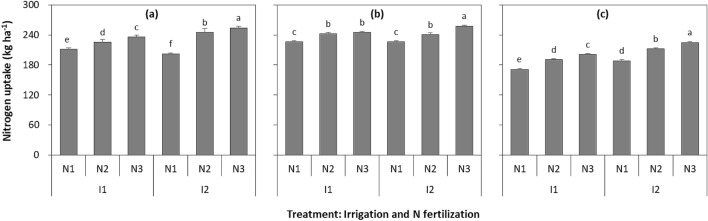
Nitrogen uptake of spring wheat during growing season with two irrigation quotas and three N fertilizer rates in (**a**) 2016, (**b**) 2017, and (**c**) 2018. I1 and I2 represent irrigation amount at 192 and 240 mm, respectively. N1, N2 and N3 represent an N fertilizer rate of 135, 180, and 225 kg N ha^−1^, respectively. Different letters indicate significant differences (*P* < 0.05) among treatments within a year and the smaller bars are standard error of means (n = 3).

Year (*P* < 0.001), irrigation quota (*P* < 0.001), N fertilizer rate (*P* < 0.001), irrigation quota × N fertilizer rate interaction (*P* < 0.001), and year × irrigation quota × N fertilizer rate interaction (*P* < 0.001) all significantly affected N-uptake of wheat (Fig. 4). In 2016, comparing N1 with N2, N-uptake improved by 6.8 and 21.3% at I1 and I2, and with N3 by 11.8 and 25.6% at I1 and I2, respectively. Comparing N3 with N2, it was reduced by 4.5 and 3.4% at I1 and I2, respectively. In 2017, comparing N1 with N2, it was improved by 7.2 and 6.0% at I1 and I2, and with N3 by 8.5 and 13.9% at I1 and I2, respectively. Comparing N3 with N2, it was reduced by 6.9% at I2. Similarly, in 2018, comparing N1 with N2, it was improved by 11.4 and 12.7%, and with N3 by 17.4 and 19.4%, respectively. Comparing N3 with N2, it was reduced by 5.1 and 5.6% at I1 and I2 respectively.

### Water use characteristics

#### Water uptake efficiency

The effect of irrigation quota, irrigation quota × N fertilizer rate interaction and year × irrigation quota × N fertilizer rate interaction on WupE were all not significant. But N fertilizer rate significantly affected it (Table 3). Compared to N3, the WupE with N2 was reduced by 3.5%; while compared to N1, it was increased by 6.0%. This indicates that reducing N fertilizer rate reduces WupE.

**Table 3:** Water uptake efficiency (WupE), water utilization efficiency (WutE), and water productivity (WUE) of spring wheat during the growing season as affected by irrigation quota and N fertilizer rate in 2016–2018.

Irrigation quota^a^	N fertilizer rate^b^	WupE (%)	WutE (kg ha^−1^ mm^−1^)	WUE (kg ha^−1^ mm^−1^)
I1	N1	61.6c	29.9a	18.4bc
	N2	65.3b	30.7a	20.0a
	N3	68.0a	29.6a	20.1a
I2	N1	61.8c	26.8b	16.6d
	N2	65.5b	29.9a	19.6ab
	N3	67.5a	26.4b	17.8cd
LSD (0.05)^c^		1.2	1.7	1.1
***P* > *F***				
Year (Y)		NS	NS	NS
Irrigation quota (I)		NS	< 0.001	< 0.001
N fertilizer rate (N)		0.003	< 0.001	< 0.001
I × N		NS	0.026	0.006
Y × I × N		NS	NS	NS

^a^I1 and I2 represent irrigation amount at 192 and 240 mm, respectively.

^b^N1, N2, and N3 represent an N fertilizer rate of 135, 180, and 225 kg N ha^−1^, respectively.

^c^The LSD (0.05) and the *P* > *F* were for all the treatments in the same column, and means with different letters in the same column are significantly different at *P* < 0.05.

#### Water utilization efficiency

A significant effect of irrigation quota, N fertilizer rate, and irrigation quota × N fertilizer rate interaction affected WutE, but not by year × irrigation quota × N fertilizer rate interaction. At I1, no significant difference of WutE among three N treatments was found (Table 3). At I2, it with N2 was improved by 11.5 and 13.2% compared to N1 and N3, respectively.

#### Water productivity

The WUE was significantly affected by irrigation quota, N fertilizer rate, and irrigation quota × N fertilizer rate interaction, but not by year × irrigation quota × N fertilizer rate interaction. At I1, the WUE with N2 was improved by 8.7% compared to N1, while had no significant difference with N3 (Table 3). At I2, the WUE with N2 was improved by 17.9 and 9.7% compared to N1 and N3.

### Nitrogen use characteristics

#### Nitrogen uptake efficiency

Year, irrigation quota, N fertilizer rate, irrigation quota × N fertilizer rate interaction (except in 2018), and year × irrigation quota × N fertilizer rate interaction all significantly affected NupE of wheat (Table 3). In 2016, compared to N1, it with N2 and N3 was reduced by 8.2 and 15.8% at I1, respectively. Comparing N3 with N2, it was increased by 9.0 and 10.2% at I1 and I2, respectively. In 2017, comparing N1 with N2, it was reduced by 7.4 and 8.4% at I1 and I2, and with N3 by 17.6 and 13.5% at I1 and I2, respectively. Comparing N3 with N2, it was increased by 12.3 and 5.8% at the two irrigation treatments. In 2018, comparing N1 with N3, it was reduced by 12.0 and 10.5%; and comparing N3 with N2, it was increased by 8.5 and 7.9%, respectively.

#### Nitrogen utilization efficiency

The NutE of wheat was significantly affected by irrigation quota, N fertilizer rate, and irrigation quota × N fertilizer rate interaction, but not by year × irrigation quota × N fertilizer rate interaction. At I1, three N treatments had no significant difference (Table 4). At I2, the NutE with N2 was improved by 11.1% compared to N3. There showed no significant difference of NutE between N1 and N2.

**Table 4:** Nitrogen uptake efficiency (NupE), N utilization efficiency (NutE), N productivity (NUE), and the ratio of N uptake to water uptake (N-uptake/W-uptake) of spring wheat during the growing season as affected by irrigation quota and N fertilizer rate in 2016–2018.

Irrigation quota^a^	N fertilizer rate^b^	NupE (%)			NutE (%)	NUE (%)	N-uptake/W-uptake		
		2016	2017	2018			2016	2017	2018
I1	N1	76.9a	79.5a	63.7c	33.8ab	24.8a	0.87a	0.89a	0.83cd
	N2	70.5b	73.6b	60.8d	35.5a	24.2a	0.88a	0.87a	0.84cd
	N3	64.7c	65.5d	56.1e	35.6a	22.0b	0.85ab	0.84a	0.80d
I2	N1	73.7ab	79.6a	70.0a	31.3bc	23.4ab	0.79b	0.89a	0.90a
	N2	76.7a	72.9b	67.6b	33.9ab	24.6a	0.91a	0.84a	0.89ab
	N3	69.7b	68.9c	62.7cd	30.5c	20.4c	0.86ab	0.88a	0.86bc
LSD (0.05)^c^		3.0	1.8	1.4	2.2	1.3	0.07	–^d^	0.03
***P* > *F***									
Year (Y)		< 0.001			NS	NS	NS		
Irrigation quota (I)		0.001	0.025	< 0.001	0.001	0.002	NS	NS	NS
N fertilizer rate (N)		< 0.001	< 0.001	< 0.001	0.001	< 0.001	0.007	NS	0.002
I × N		< 0.001	0.002	NS	0.009	0.008	NS	NS	NS
Y × I × N		< 0.001			NS	NS	0.002		

^a^I1 and I2 represent irrigation amount at 192 and 240 mm, respectively.

^b^N1, N2, and N3 represent an N fertilizer rate of 135, 180, and 225 kg N ha^−1^, respectively.

^c^The LSD (0.05) and the *P* > *F* were for all the treatments in the same column, and means with different letters in the same column are significantly different at *P* < 0.05.

^d^LSD not provided when the corresponding *P* > *F* from analysis of variance is not significant at *P* ≤ 0.05.

#### Nitrogen productivity

A significant effect of irrigation quota, N fertilizer rate, and irrigation quota × N fertilizer rate interaction affected NUE, but not by year × irrigation quota × N fertilizer rate interaction. At I1, the NUE with N2 was improved by 9.8% compared to N3 (Table 4). Similarly, at I2, it was improved by 20.4%. No significant difference of NUE between N1 and N2 were revealed.

### Ratio of nitrogen uptake to water uptake

**Figure 5:**
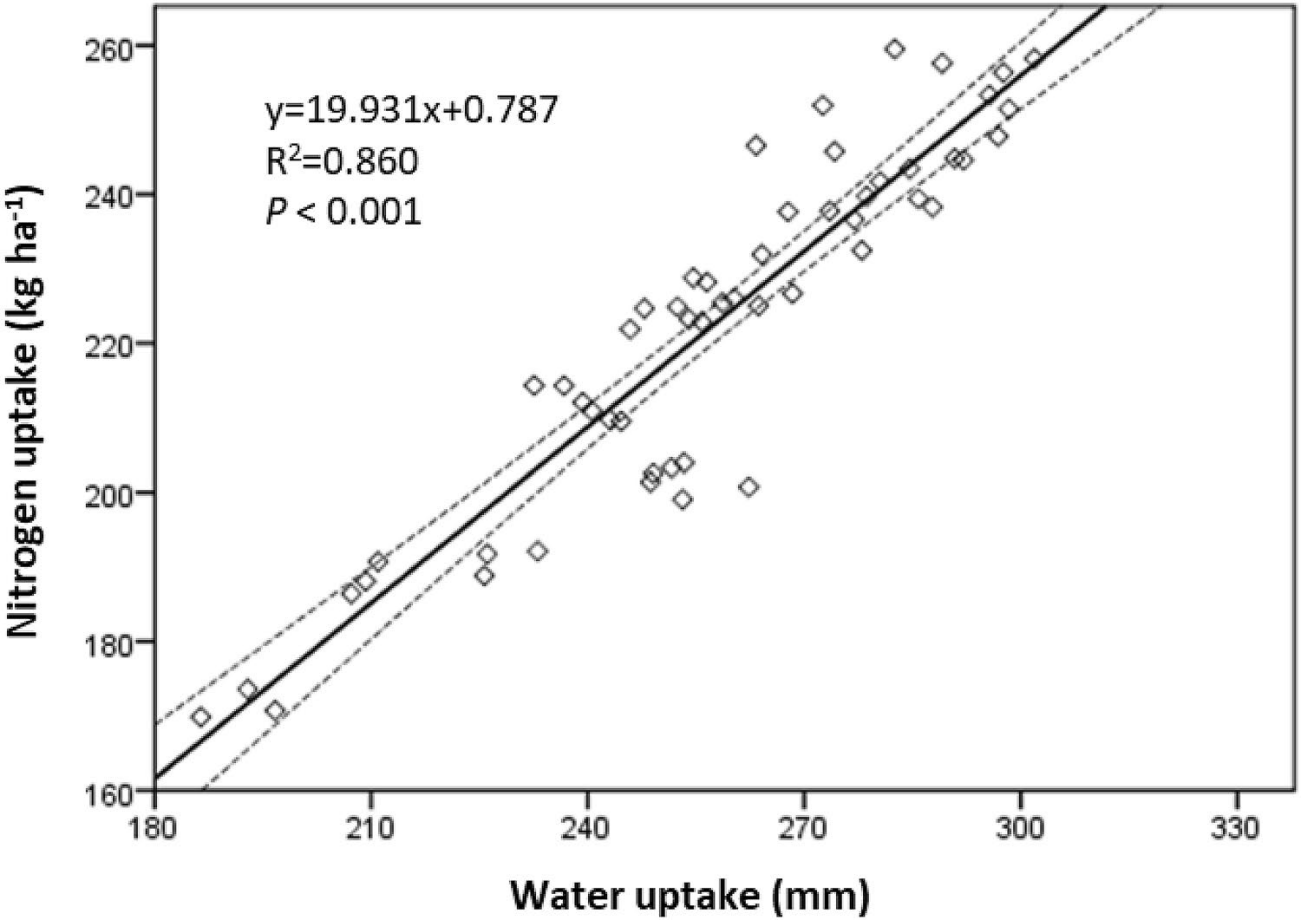
The relationship between water uptake and nitrogen uptake of spring wheat during the growing season in 2016–2018. The solid line and medium dash lines indicate the regression line and 95% confidence intervals.

The ratio of N-uptake to W-uptake was significantly affected by irrigation quota (except in 2016 and 2017), N fertilizer rate, irrigation quota × N fertilizer rate interaction (except in 2018), and year × irrigation quota × N fertilizer rate interaction (*P* = 0.002). In 2016, comparing N1, the ratio of N-uptake to W-uptake with N2 was improved by 14.5% at I2 (Table 4). In 2017, no significant difference of ratio of N-uptake to W-uptake was found among any treatments. In 2018, comparing N1 with N3, it was reduced by 4.1% at I2. The results indicate that the ratio of N-uptake to W-uptake was constant and interrelated with each other. This was evidenced by a further regression analysis that W-uptake and N-uptake followed in a linear regression curve (Fig. 5).

### Path and regression analysis

**Figure 6:**
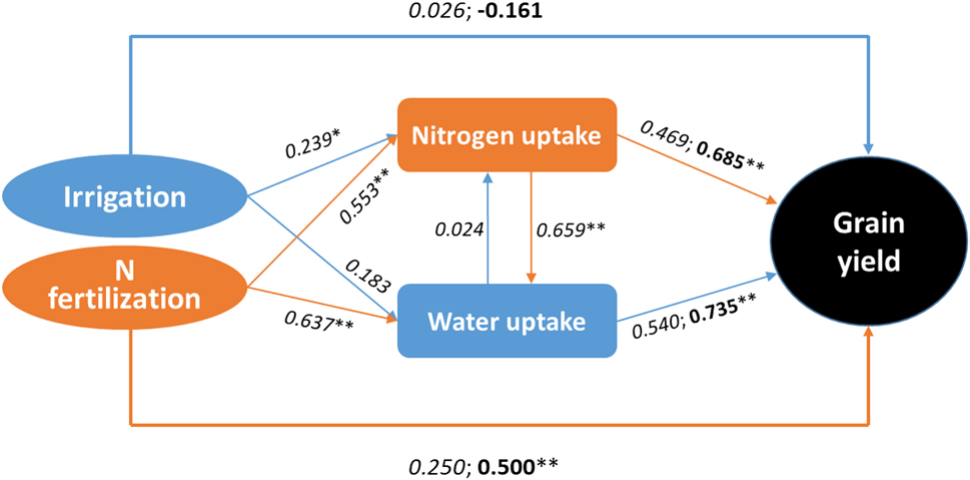
Path analysis of irrigation and N fertilization on grain yield of spring wheat via influence on nitrogen uptake and water uptake across 2016–2018. Fine lines represent indirect pathways and thick lines represent direct pathways. Italicized values are the path coefficient and bold values are the correlation coefficient. *P ≤ 0.05, ** *P* ≤ 0.01.

The path analysis revealed that GY had a significant positive correlation with W-uptake and N-uptake (Fig. 6). The W-uptake explained 54.0% of GY, while N-uptake explained 46.9%. In more detail, irrigation quota had a direct negative effect on GY, but it positively and indirectly affected GY through N-uptake and W-uptake, with N-uptake contributing more. In contrast, N fertilizer rate had a direct positive effect on GY, also it indirectly affected GY through N-uptake and W-uptake, with W-uptake contributing more. A significant direct influence of N-uptake via W-uptake indicated the interaction-effect between irrigation and N fertilization.

**Figure 7:**
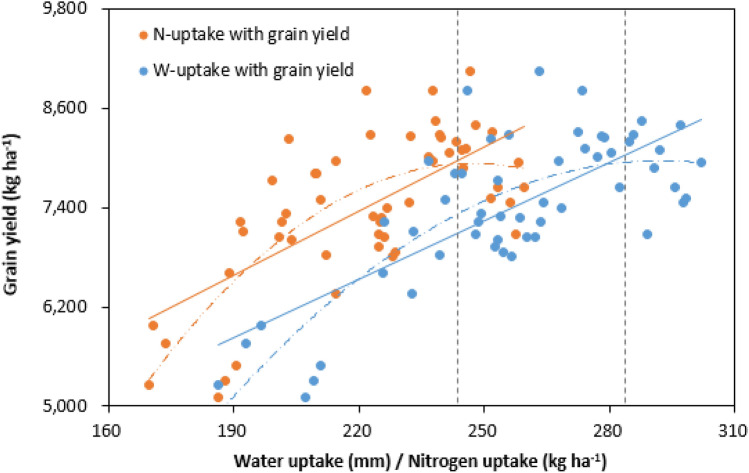
The relationship between water uptake and grain yield (blue circles and lines) and nitrogen uptake and grain yield (orange circles and lines) of spring wheat during the growing season in 2016–2018. The dash lines indicate the values of water uptake or nitrogen uptake when grain yield reaches the highest in a quadratic function.

The regression analysis showed that both W-uptake and N-uptake closely related with grain yield (Fig. 7). They both followed in a primary liner regression curve and a quadratic liner regression curve. Whereas, the quadratic function with R^2^ value greater than primary function, meaning that quadratic function explained greater to the variation of their correlation (Table 5). Besides, the *F* value of quadratic function was lower than that of primary function, indicating quadratic function was more suitable for modeling relationship of W-uptake and N-uptake with GY. It is obvious that W-uptake was the key determinants for achieving the highest GY (Fig. 7). However, with increase of W-uptake or N-uptake, GY cannot consistently increase. The proper value of W-uptake and N-uptake for achieving the highest GY were 292 mm and 247 kg N ha^−1^, respectively, according to quadratic functions.

**Table 5:** Regression of grain yield (GY) with nitrogen uptake (N-uptake) and water uptake (W-uptake) of spring wheat at Wuwei Experimental station across 2016–2018.

Regression variable	Equation type	Model summary			Parameter estimation^a^		
		R^2^	*F*	*P*	b1	b2	b3
GY and W-uptake	Primary function	0.540	61.0	< 0.001	–	23.60	1348.19
	Quadratic function	0.627	42.8	< 0.001	− 0.273	159.65	− 15,357.41
GY and N-uptake	Primary function	0.469	46.0	< 0.001	–	25.95	1650.49
	Quadratic function	0.553	31.5	< 0.001	− 0.436	215.48	− 18,696.83

^a^b1 is the coefficient of quadratic term, b2 is the coefficient of primary term, and b3 is the constant term.

## Discussion

### Efficient use of soil water

Water is a primary resource for plant growth, which also provide dissolution of nutrients for crop requirement^35^. Therefore, optimizing root environment and suppling of additional water often increases wheat yield^17,36^. Nevertheless, consistently supply of additional water cannot always increase the grain yield, as some of the water may consume invalidly through soil evaporation, expecially in arid conditions^12,37^. This implies that any efforts on yield improvement should also focusing on invalid water reduction. The direct way is to improve the capacity of crops for uptake water from soil layers^17,38,39^. In the present study, W-uptake was quantified as total water consumption minus the soil evaporation (with neglect of canopy evaporation). Generally, an increase of irrigation quota can increase (by 4.1%) W-uptake of wheat. However, increasing of irrigation quota did not promote the WupE. The possible reason may be that separating of irrigation at seedling, booting and grain filling stage might mask the influence of irrigation on wheat growth. In addition, more irrigation water often generate more invalid water loss through soil evaporation^16^. It obvious that increase irrigation decreased the WutE. Compared to irrigation at 192 mm (I1), the WutE of spring wheat with irrigation at 240 mm (I2) was lowered by an average of 8.7%. A significant reduction in dry matter re-distribution was mainly attributed to the lowered WutE^40^. As dry matter allocation from vegetative organs to grains usually promoted with water limited conditions^41^. Hence, GY of wheat with I1 was increased by 6.3 and 7.9% at N fertilizer rate of 135 kg N ha^−1^ (N1) and 225 kg N ha^−1^ (N3), compared to I2. Accordingly, WUE with I1 was increased by 11.0 and 12.8% at N1 and N3, compared to I2.

Commonly, fertilization improves the amount of water extracted by crops from deeper soil layers^42^. In the present study, W-uptake by wheat was closely related to N fertilizer rate. The more N fertilizer input, the more W-uptake. Compared to N1, W-uptake with N2 was increased by 10.3%; while compared to N3, it with N2 was reduced by 7.0%. Also, increasing the N fertilizer rate increased the WupE. Comparing N1, WupE with N2 was increased by 6.0%; while comparing N3 with N2, it was reduced by 3.5%. This mainly because of the enlarged canopy with high N rate, which reduces soil evaporation^39,43^. However, increasing N fertilizer rate did not promote the WutE. On the contrary, the more N fertilizer input, the lower water was utilized by wheat to form the grains. Compared to N3, WutE with N2 was increased by 13.2%. This mainly because that sufficient soil available N may generate vigorous vegetative growth, which delays the reproductive growth^32^. Besides, N remobilization from vegetative to reproductive organs was more efficient at low soil N conditions^44^. However, a lower dose of N application may reduce the growth of crops, which depress the water extraction from soil and reduce the grain yield^43^. Therefore, compared to N1, WutE with N2 was increased by 11.5%. Moreover, the GY with N2 was improved by 17.8% compare to N1. As a consequence, WUE with N2 was improved by 17.9% compare to N1.

### Efficient use of soil nitrogen

Nitrogen as a critical element of plant proteins, is essential to development of crop growth and compound for grain yield^35^. Hence, improving N use efficiency was widely recognized as a priority to achieve higher net income and better environmental effect^20^. Several studies have reported that irrigation amounts had significant effect on N use efficiency^17,45^. Timsina et al.^46^ reported that irrigation could improve the agronomic N use efficiency, physiological efficiency, and fertilizer N recovery efficiency of crops. However, in irrigation areas, a deficit irrigation was always used to promote N uptake and N use efficiency^47^. Because lower irrigation is more effective at reducing N leaching^48^. In this study, irrigation with higher amount constantly had greater N-uptake than lower irrigation when N fertilizer rate was higher (with N2 and N3). Also, the NupE was greater with higher irrigation than lower irrigation. Whereas, the NutE (by 16.8%) and NUE (by 7.9%) were significantly reduced with higher irrigation compared to lower irrigation at highest N fertilizer rate (N3); and remaining no significant difference when N fertilizer rate was lower (with N1 and N2). This indicated that higher irrigation increased the opportunity of N fertilizer to be dissolved in irrigation water and leached deeper into the soil^48^. While, lower irrigation maintain the fertilizer distributed within the upper soil layers and available for wheat roots, thereby increase the NUE^47^.

Commonly, increasing N fertilizer rate negatively affected NUE^21,46^. Although the ability of a crop to extract soil N might be increased with more N input, the convertion of absorbed N into harvest grains is always lowered^44^. In the present study, N-uptake of spring wheat was significantly improved with increasing of N fertilizer rate (except with I1 in 2017). However, the NupE was significantly reduced with increasing of N rate (except with I2 in 2016). The possible reason are (1) fertilizer N was base applied before planting, which lowered the synchrony between crop N demand and supply throughout the growing season^49^, and (2) a greater fertilizer N supply rather than indigenous N may increase the potential for N losses^50^. Furthermore, increasing N fertilizer rate would lower the N remobilization, leading to less N transfer from vegetative to reproductive organs^51^. As a result, the NutE efficiency with N2 was increased by 11.1% compared to N3 at I2. This indicated that lower irrigation could lessen the negative effect of N rate on N remobilization. However, it can hardly remove the negative effect, as NUE with N2 was increased by 9.8% compared to N3 at I1. With higher irrigation, the NUE improved even greatly (by 20.4%). However, the present research missed the determination of soil NH_4_–N and NO_3_–N concentrations in different soil layers, which are essential for addressing the underlying mechanisms for soil N use. A further study concerning N leaching and N balance is fundamentally required.

### Mechanisms on yield improvement of spring wheat

It has been reported that there was an interaction between water and N fertilizer^52^. An appropriate soil water level had a positive effect on uptake of soil N, and an appropriate N fertilizer rate promoted the use of soil water^3^. In the present study, a relative stable value of ratio of N-uptake to W-uptake was revealed. Besides, the two indicators followed in a linear regression curve. These results indicating an obvious interaction effect. We conducted a further pathway analysis and found a significant indirect effect of N-uptake on GY via W-uptake. Besides, irrigation affected GY through N-uptake, while N fertilization affected GY mainly through W-uptake. This may be the underlying reason for the conjunction, i.e. the interaction effect between irrigation and N fertilization. Because increasing N-uptake must rely on the increase of W-uptake, while increasing of W-uptake was due to higher transpiration. Therefore, the differences in wheat yield was better explained by differences in water use rather than N use^21^. The W-uptake should be the primary limiting factor for achieving higher yield^17^. According to path analysis, W-uptake was only determined by N fertilization but not irrigation, as path coefficient of irrigation to W-uptake was not significant. Hence, much attention should be focused on management of N fertilization rather than irrigation in wheat production in this irrigation region.

In terms of yield responses to total N-uptake, several researchers have reported marked increases of wheat yield with increasing of N-uptake^17,53,54^. In this study, an increase of GY with increasing of N-uptake was observed when N-uptake value was lower than 247 kg N ha^−1^, while it decreased when the value exceeds than 247 kg N ha^−1^. Similarly, there was a significant correlation in wheat yield with W-uptake, and more W-uptake signify higher yield^55^. In this study, an increase of GY with increasing of W-uptake was observed when W-uptake value lower than 292 mm, while decreased with the value exceeds than 292 mm. This mechanism of high-yield inspired us that we should manage proper irrigation quota and N fertilizer rate to optimize W-uptake and N-uptake in wheat production to increase GY.

## Conclusion

Reducing irrigation quota from 240 to 192 mm coupled with N fertilizer reduction from 225 to 180 kg N ha^−1^ maintained the same quantity of GY in comparison to local practices. This mainly attributable to enhanced WutE and WUE, and promoted NupE, NutE and NUE. While, it reduced W-uptake and WupE, and lowered N-uptake. A strong liner correlation and significant path coefficient between N-uptake and W-uptake revealed the underlying mechanism on interaction between irrigation and N fertilization. Moreover, N fertilization management was rather important than irrigation for wheat production in irrigation region. This kind of scientific finding may provide a clear way for achieving of a water-saving and N-saving farming model, thereby to sustainably produce wheat in arid areas. Further researches that can accurately simulation yield potential with lowest irrigation and N fertilization are urgently needed, so that improve the economic and ecological benefits in crop production.

## Supplementary Information

Supplementary Information.
