# Supplementary material for: Improving wheat grain yield via promotion of water and nitrogen utilization in arid areas
Source: Sci Rep. 2021 Jul 5;11:13821. doi: 10.1038/s41598-021-92894-6 (PMC8257629; doi:10.1038/s41598-021-92894-6)
Supplement: Supplementary file 1 — Supplementary Information. [file 41598_2021_92894_MOESM1_ESM.docx]

**Table S-1.** Grain yield (GY) and water uptake (W-uptake) of spring wheat as affected by irrigation quota and N fertilizer rate in 2016-2018.

| Irrigation | N Fertilizer | GY (kg ha^-1^) | | |  | W-uptake (mm) | | |
| --- | --- | --- | --- | --- | --- | --- | --- | --- |
| quota ^a^ | rate ^b^ | 2016 | 2017 | 2018 |  | 2016 | 2017 | 2018 |
| I1 | N1 | 7866 | 7060 | 5664 |  | 241 | 254 | 192 |
|  | N2 | 8189 | 8141 | 6985 |  | 255 | 278 | 228 |
|  | N3 | 8227 | 8238 | 7727 |  | 278 | 291 | 251 |
| I2 | N1 | 7132 | 6945 | 5299 |  | 255 | 256 | 209 |
|  | N2 | 8731 | 8062 | 6896 |  | 270 | 285 | 237 |
|  | N3 | 7416 | 7685 | 7312 |  | 294 | 294 | 262 |
| *P* > *F* ^c^ | | | | | | | | |
| Year (Y) |  | < 0.001 |  |  |  | 0.635 |  |  |
| Irrigation quota (I) | | 0.006 |  |  |  | < 0.001 |  |  |
| N Fertilizer rate (N) | | < 0.001 |  |  |  | < 0.001 |  |  |
| I × N |  | 0.014 |  |  |  | 0.987 |  |  |
| Y × I × N |  | 0.132 |  |  |  | 0.707 |  |  |
| ^a^ I1 and I2 represent irrigation amount at 190 and 240 mm, respectively.  ^b^ N1, N2, and N3 represent an N fertilizer rate of 135, 180, and 225 kg N ha^-1^, respectively.  ^c^ The *P* > *F* were for all the treatments of each indicator in three study years. | | | | | | | | |
